# Supplementary material for: Full Vectorial Field Sensing Using Liquid Crystal Droplet Arrays
Source: Adv Mater. 2026 Jun 9;38(39):e73661. doi: 10.1002/adma.73661 (PMC13361162; doi:10.1002/adma.73661)
Supplement: Supplementary file 1 — Supporting File: adma73661‐sup‐0001‐SuppMat.docx. [file ADMA-38-e73661-s001.docx]

**Supplementary Notes: Full vectorial field sensing using liquid crystal droplet arrays**

Xuke Qiu^†^, Jinge Guo^†^, Jiahe Cui, Runchen Zhang, Zimo Zhao, Yifei Ma, Waqas Kamal, Steve J. Elston, Alfonso A. Castrejón-pita, Stephen M. Morris^*^, and Chao He^*^

*Department of Engineering Science, University of Oxford, Parks Road, Oxford, OX1 3PJ, UK*

^†^*These authors contributed equally to this work*

*^*^Corresponding authors:* [*stephen.morris@eng.ox.ac.uk*](mailto:stephen.morris@eng.ox.ac.uk)*;* [*chao.he@eng.ox.ac.uk*](mailto:chao.he@eng.ox.ac.uk)

**Supplementary Note 1: Stokes polarimeter and LC droplet characterization**

Our system determines the states of polarization (SoP) of light using the Stokes polarimetry principle, in which the SoP is represented by a four-component Stokes vector: $S_{0}$, $S_{1}$, $S_{2}$, and $S_{3}$. Together, these components provide a complete description of the light’s polarization characteristics, encompassing total intensity, linear polarization along orthogonal axes, and circular polarization.

To determine the four Stokes components, at least four independent intensity measurements are required, typically obtained by analysing light after it passes through different polarization filters. Depending on the measurement strategy, Stokes polarimeters can be classified into time-sequential and simultaneous types. In the time-sequential approach, light is analysed successively through a series of polarization elements – such as linear polarizers and wave plates – each isolating one Stokes parameter. Although conceptually simple, this method demands multiple exposures and is unsuitable for dynamic light fields. In contrast, simultaneous polarimetry acquires all Stokes parameters in a single shot by using multiple detector arrays or specially designed optical configurations. A representative implementation is the division-of-wavefront polarimetry (DoWP), which splits the incident beam into sub-wavefronts that traverse different polarization channels, enabling the concurrent retrieval of all Stokes parameters.

In our system, we utilize LC droplets to measure the SoP of light using the DoWP method. The LC droplets serve as polarization-sensitive elements, enabling the simultaneous analysis of the light's polarization state. By employing a mueller matrix polarimeter, we can characterize the patterns of retardance (the phase difference between the ordinary and extraordinary rays) and the orientation of the fast axis (the axis along which the light experiences the least phase delay) within the LC droplets we use as shown in **Supplementary Figure 1**.

The retardance pattern of the LC droplets reveals concentric circles, where the retardance increases from the centre outward and then resets ($0^{\circ}-180^{\circ}$). The fast axis orientation shows radial symmetry, indicating that each position of the droplet modulates the polarization state of the incident light differently. As a result, when uniform polarized light illuminates the droplet, each segment of the emitted light experiences distinct modulation effects based on its position relative to the droplet’s centre. This behaviour allows each point on a droplet to function as a tiny waveplate, each with its own retardance and fast axis orientation. Thus, a single LC droplet effectively operates as a DoWP, enabling the simultaneous capture of all Stokes parameters across the wavefront.

A limitation of the DoWP is that it can only measure the SoP at a single spatial location, yielding a single SoP result. In other words, a single LC droplet can only detect the SoP of the light beam within its coverage area. To achieve spatially resolved measurements, an array of LC droplets is employed, where each droplet independently analyses the local polarization state. This configuration allows the LC droplet array polarimeter to measure spatially and temporally varying SoPs in real time.

Based on this concept, we developed an LC droplet array polarimeter. The setup begins with a laser light source, which passes through a polarization state generator (PSG) composed of a polarizer and a quarter-wave plate (QWP). This arrangement generates various SoPs, simulating demanded input polarizations for the measurement process. The generated light then enters our LC droplet array polarimeter, which consists of an LC droplet array, a polarizer, and a CCD camera.

We define the Stokes vector of the incident light as $S_{in} =\left( S_{0,in},S_{1,in},S_{2,in},S_{3,in} \right)^{T}$. After passing through the LC droplet array polarimeter and being received by the CCD camera, the output light is defined as $S_{out}=\left( S_{0,out},S_{1,out},S_{2,out},S_{3,out} \right)^{T}$. The relationship between the incident and output Stokes vectors can be expressed as:

$$\begin{aligned} S_{out}=M_{P}\cdot M_{LC}\cdot S_{in}\#\left( 1 \right) \end{aligned}$$

Here, $M_{P}$ and $M_{LC}$ represent the Mueller matrices (MM) of the polarizer and the LC droplet array, respectively. Since the CCD camera can only capture the intensity information of the light, corresponding to the first element of $S_{out}$ ($S_{0,out}$), so only the first row of the Mueller matrices $M_{P}$ is relevant. The first row of the Mueller matrices for a linear polarizer can be expressed as:

$$\begin{aligned} M_{P}\left( 1,: \right)=\left( \begin{matrix} p_{t}, & p_{t}, & 0, & 0 \end{matrix} \right).\#\left( 2 \right) \end{aligned}$$

As mentioned earlier, the LC droplet can be modelled as comprising $n$ micro-waveplates, each with its own Mueller matrix $M_{n, LC}$, expressed as:

$$\begin{aligned} M_{n, LC}\approx\left[ \begin{matrix} 1, & 0, & 0, & 0 \\ 0, & \cos^{2}2\theta_{n}+\sin^{2}2\theta_{n}cos\delta_{n}, & 0.5sin4\theta_{n}\left( 1-cos\delta_{n} \right), & -sin2\theta_{n}\sin\delta_{n} \\ 0, & 0.5sin4\theta_{n}\left( 1-cos\delta_{n} \right), & \sin^{2}2\theta_{n}+\cos^{2}2\theta_{n}\cos\delta_{n}, & cos2\theta_{n}\sin\delta_{n} \\ 0, & sin2\theta_{n}\sin\delta_{n}, & -cos2\theta_{n}\sin\delta_{n}, & \cos\delta_{n} \end{matrix} \right],\#\left( 3 \right) \end{aligned}$$

where $\theta_{n}$ and $\delta_{n}$ represent the fast axis direction and retardance of the $n^{th}$ tiny wave plate. In real experiment, the CCD camera simultaneously captures the intensity values corresponding to the output of each tiny waveplate, denoted as $S_{0n, out}$. By substituting Eq.2 and Eq.3 into Eq.1, we derive:

$$\begin{aligned} S_{0n, out}=\left( \begin{matrix} p_{t}, & p_{t}\left( \cos^{2}2\theta_{n}+\sin^{2}2\theta_{n}\cos\delta_{n} \right), & 0.5p_{t}sin4\theta_{n}\left( 1-cos\delta_{n} \right), & -p_{t}sin2\theta_{n}\sin\delta_{n} \end{matrix} \right)\cdot\left( \begin{matrix} S_{0,in} \\ S_{1,in} \\ S_{2,in} \\ S_{3,in} \end{matrix} \right).\#\left( 4 \right) \end{aligned}$$

Finally, by combining the results from the $n$ measurements, we obtain:

$$\begin{aligned} I=\left( \begin{matrix} p_{t}, & p_{t}\left( \cos^{2}2\theta_{1}+\sin^{2}2\theta_{1}\cos\delta_{1} \right), & 0.5p_{t}sin4\theta_{1}\left( 1-cos\delta_{1} \right), & -p_{t}sin2\theta_{1}\sin\delta_{1} \\ \vdots& \vdots& \vdots& \vdots\\ p_{t}, & p_{t}\left( \cos^{2}2\theta_{n}+\sin^{2}2\theta_{n}\cos\delta_{n} \right), & 0.5p_{t}sin4\theta_{n}\left( 1-cos\delta_{n} \right), & -p_{t}sin2\theta_{n}\sin\delta_{n} \end{matrix} \right)\cdot\left( \begin{matrix} S_{0,in} \\ S_{1,in} \\ S_{2,in} \\ S_{3,in} \end{matrix} \right),\#\left( 5 \right) \end{aligned}$$

where $I$ is an $n\times1$ array, representing all the light intensities captured by the CCD camera for each point on the droplet array. Here, $n$ corresponds to the resolution of the CCD camera. We can now express the bulk matrix in Eq.5 as $A$, an $n\times4$ matrix. This matrix $A$ represents the system Mueller matrix of the entire LC droplet array polarimeter. In other words, as long as the LC droplet array and the polarizer remain unchanged, the system matrix $A$ will remain constant. Consequently, the relationship can be expressed as:

$$\begin{aligned} I=A\cdot S_{in}\#\left( 6 \right) \end{aligned}$$

The objective is to determine the incident Stokes vector $S_{in}$. While the intensity vector $I$ is directly obtained from the CCD camera, solving for $S_{in}$ requires an accurate calibration of the system matrix $A$, performed using Mueller matrix polarimetry. The relationship defining$A$ for the LC droplet array polarimeter follows Eq. (6) and can be expressed as:

$$\begin{aligned} A=I\cdot{S_{in}}^{\dagger},\#\left( 7 \right) \end{aligned}$$

where ${S_{in}}^{\dagger}$ represents the pseudoinverse of $S_{in}$. Typically, in Mueller matrix polarimetry, the output Stokes vector $S_{out}$ is related to the incident Stokes vector $S_{in}$ through equation $S_{out}=M\cdot S_{in}$. In conventional Mueller‑matrix polarimetry, one fixes the input polarization and then analyses the emerging $S_{out}$ by rotating elements in a polarization state analyser (PSA)-typically a QWP and polarizer-to sample different output projections. This approach requires a mechanically rotating analyser in the detection path, and each new analyser setting constitutes a separate measurement of the same unknown state. By contrast, our calibration strategy keeps the detection path completely static (no PSA rotations are needed) and instead varies the input polarization states $S_{in}$ at the source.

This method enables calibration of the system matrix $A$ within the same optical configuration. A set of linearly independent input polarization states was generated using the PSG. To improve calibration reliability and reduce measurement uncertainty, we employed 72 different polarization states for calibration. Specifically, the first set of 36 polarization states was generated by fixing the polarizer at $0^{\circ}$ and rotating QWP from $0^{\circ}$ to ${180}^{\circ}$ in $5^{\circ}$ increments. Subsequently, the QWP was removed, and the polarizer was rotated from $0^{\circ}$ to ${180}^{\circ}$, also in $5^{\circ}$ increments, generating additional 36 polarization states. For each of the 72 polarization states, the intensity values of the output light were recorded by the CCD camera, providing a total of $n\times72$ intensity measurements that form the matrix $I$. Concurrently, the Stokes vectors corresponding to these 72 polarization states were combined into a $4\times72$ matrix representing $S_{in}$. By substituting these values into Eq.7, we compute the system matrix $A$, which is of size $n\times4$. The system matrix $A$ is invariant with respect to the incident light and remains fixed as long as the configuration of the LC droplet array and the polarizer is unchanged. Once $A$ has been calibrated, the LC droplet array polarimeter can accurately retrieve the Stokes vector $S_{in}$ of any unknown incident light. This is achieved by rearranging Eq.6 to express $S_{in}$ as:

$$\begin{aligned} S_{in}=A^{\dagger}\cdot I,\#\left( 8 \right) \end{aligned}$$

where the measured intensity $I$ and the inverse of the calibrated system matrix $A$ are used to solve for $S_{in}$.

In addition to polarization information, the intensity information is directly obtained from the same CCD images used for Stokes reconstruction. Each droplet region on the CCD provides an effective measurement channel, with its mean transmitted intensity extracted to form the corresponding element of the intensity vector $I$. A reference frame recorded without the analyser is used to normalize illumination and correct for spatial nonuniformity. This procedure yields calibrated intensity data consistent with the system matrix model, enabling simultaneous acquisition of total intensity and polarization information without additional optics or sequential steps.


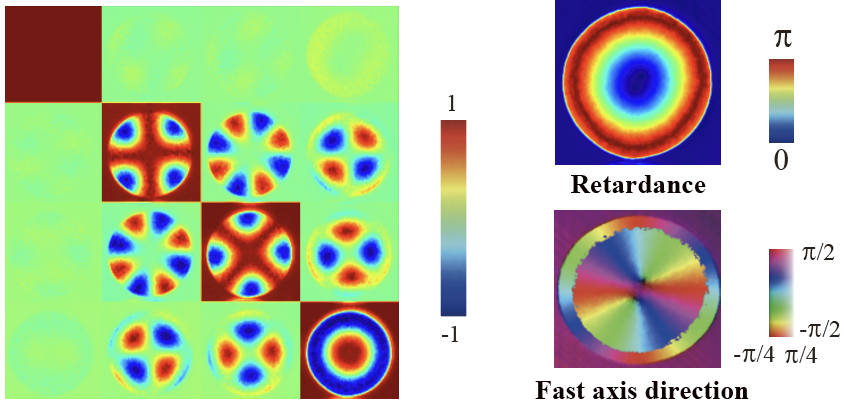


**Supplementary Figure 1 Optical characterization of the LC droplet through its Mueller matrix and decomposition into retardance and fast axis orientation profiles.** The Mueller matrix provides a complete description of the droplet’s optical response. Retardance exhibits concentric rings, with values increasing from the centre, peaking around the midpoint radius, and decreasing to zero near the edge. The fast axis orientation shows radial symmetry, varying smoothly with angular position around the droplet centre. The fast axis is represented using the Hue-Saturation-Luminance model, where hue denotes the axis orientation (from $-\pi/2$ to $\pi/2$), luminance reflects ellipticity (from $-\pi/4$ to $\pi/4$), and saturation is fixed at 1. Retardance values range from 0 to $\pi$, as indicated by the color bar.

**Supplementary Note 2: Shack-Hartmann wavefront sensor**

In this study, the LC droplet array is employed as the lenslet array of a Shack**-**Hartmann wavefront sensor (SHWS) to reconstruct the wavefront of the optical beam. The droplets divide the incident wavefront into multiple portions, with each droplet serving as an individual focusing element. The transmitted light is focused onto a CCD, forming an array of focal spots, one spot behind each droplet, referred to as Shack-Hartmann spots. For an ideal, aberration-free wavefront, these spots form a regular grid, whereas wavefront aberrations introduce displacements from their reference positions, from which local wavefront slopes are determined (**Supplementary Figure 2(a)**).

To reconstruct the complete wavefront, Zernike polynomials are utilized because they are particularly well-suited for representing wavefront distortions in optical systems, especially when dealing with circular apertures, such as the ones defined by the LC droplets in this configuration. Zernike polynomials can effectively capture various types of optical aberrations (e.g. defocus, astigmatism, and coma) with a compact set of orthogonal mathematical functions. The wavefront $\Phi\left( \rho,\theta\right)$ is expressed as:

$$\begin{aligned} \Phi\left( \rho,\theta\right)=\sum_{i=2}^{N} a_{i}Z_{i}\left( \rho,\theta\right)\#\left( 9 \right) \end{aligned}$$

where $a_{i}$ is the coefficient of the $i^{th}$ Zernike polynomial $Z_{i}\left( \rho,\theta\right)$, representing its RMS phase value. The coefficients $a_{i}$ can be obtained using the orthogonality of Zernike polynomials,

$$\begin{aligned} a_{i}=\frac{1}{\pi}\int_{0}^{1} \int_{0}^{2\pi} \Phi\left( \rho,\theta\right)Z_{i}\left( \rho,\theta\right)d\rho d\theta.\#\left( 10 \right) \end{aligned}$$

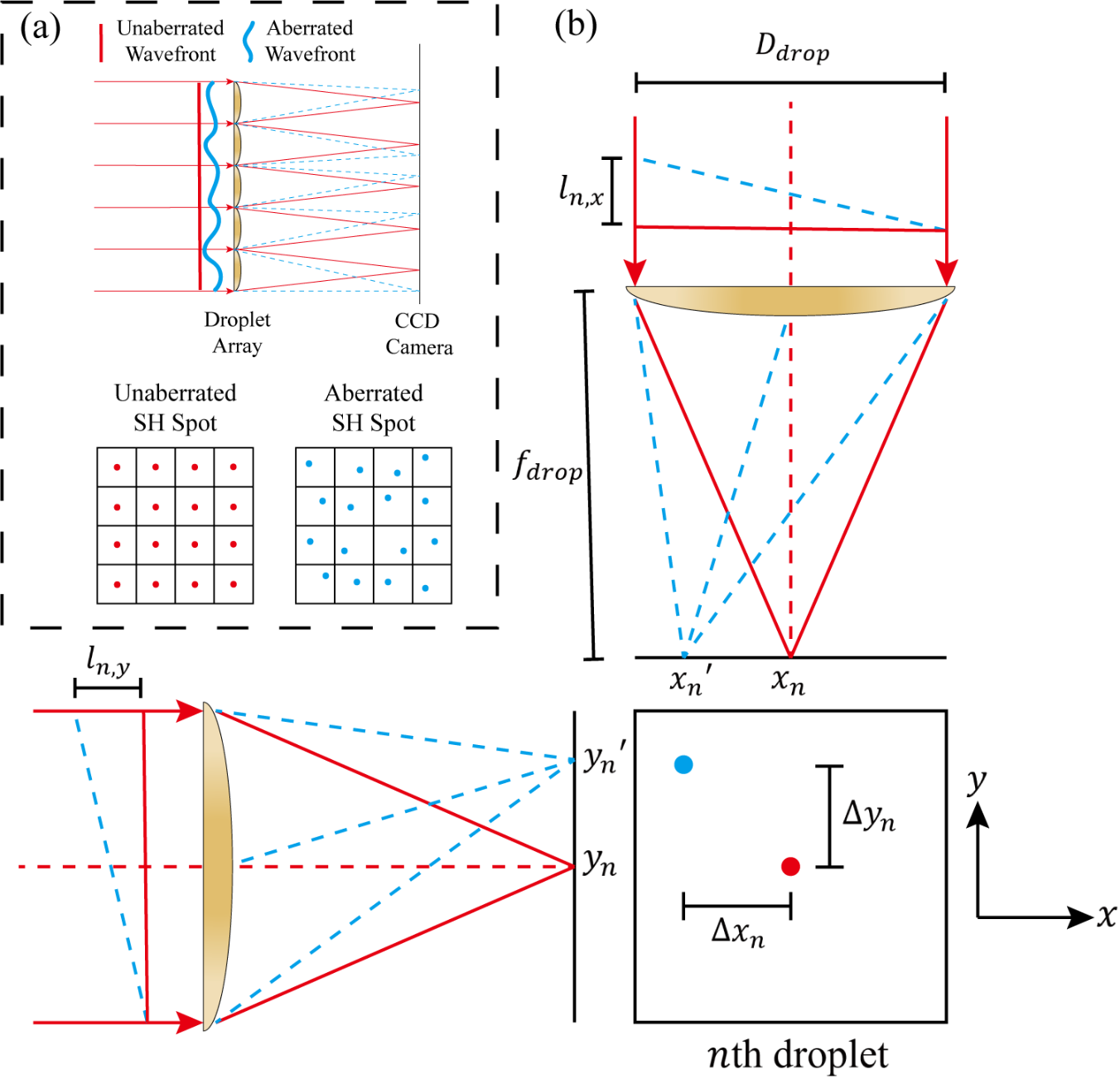


**Supplementary Figure 2 This diagram presents an overview of the SHWS configuration along with the corresponding focal spot pattern.** (a) The spot pattern: an ideal wavefront produces a uniform array of spots, whereas an aberrated wavefront results in shifted spots that reveal the local wavefront tilt. (b) The sensor geometry: a collimated beam is divided by an array of droplets, with each droplet sampling a small portion of the wavefront The local tilt of the wavefront causes a displacement of the focal spot along the *x* and *y* axes.

The precise position of each Shack-Hartmann spot is calculated using a centroiding algorithm that computes the centre of mass of the intensity distribution of each focal spot. The centroid coordinates $(x_{c}, y_{c})$ are determined by:

$$\begin{aligned} x_{c}=\frac{\sum_{m=1}^{N} x_{m}I_{m}}{\sum_{m=1}^{N} I_{m}}, y_{c}=\frac{\sum_{m=1}^{N} y_{m}I_{m}}{\sum_{m=1}^{N} I_{m}}\#\left( 11 \right) \end{aligned}$$

where $x_{m}$ and $y_{m}$ are the coordinates of the $m^{th}$ pixel, and $I_{m}$ is the intensity at that pixel. By comparing the centroid positions with the expected positions for an unaberrated wavefront, the displacements $\Delta x$ and $\Delta y$ are calculated (**Supplementary Figure 2(b)**). These displacements are directly proportional to the local wavefront slopes:

$$\begin{aligned} \frac{\partial\left( \Phi_{n} \right)}{\partial x}\simeq\frac{l_{n,x}}{D_{drop}}=\frac{\Delta x_{n}}{f_{drop}}, \frac{\partial\left( \Phi_{n} \right)}{\partial y}\simeq\frac{l_{n,y}}{D_{drop}}=\frac{\Delta y_{n}}{f_{drop}}\#\left( 12 \right) \end{aligned}$$

where $f_{drop}$ represents the equivalent focal length of the LC droplets, and $D_{drop}$ is the diameter of the droplet. $l_{n,x}$ and $l_{n,y}$ are the optical path differences (OPD) in the $x$ and $y$ directions.

Since the CCD camera records the intensity on a rectangular grid, the phase aberration $\Phi$ is also represented in Cartesian coordinates by rewriting Eq.9 as

$$\begin{aligned} \Phi\left( x,y \right)=\sum_{n=2}^{N} a_{n}Z_{n}\left( x,y \right).\#\left( 13 \right) \end{aligned}$$

with $\rho=\sqrt{x^{2}+y^{2}}$ and $\theta=\tan\frac{y}{x}$. Assume $(x_{n},y_{n})$ are now normalized coordinates of the centre of the $n^{th}$ droplet within a unit circle, the local wavefront tilt at that droplet is expressed as

$$\begin{aligned} \frac{\partial\left( \Phi_{n}\left( x_{n},y_{n} \right) \right)}{\partial x}=\frac{\Delta x_{n}}{f_{drop}}=\sum_{i=2}^{N} a_{i}\bar{\frac{{\partial Z}_{i}\left( x_{n},y_{n} \right)}{\partial x}},\#\left( 14 \right) \end{aligned}$$

$$\begin{aligned} \frac{\partial\left( \Phi_{n}\left( x_{n},y_{n} \right) \right)}{\partial y}=\frac{\Delta y_{n}}{f_{drop}}=\sum_{i=2}^{N} a_{i}\bar{\frac{{\partial Z}_{i}\left( x_{n},y_{n} \right)}{\partial y}}\#\left( 15 \right) \end{aligned}$$

where $\bar{\frac{{\partial Z}_{i}(x_{n},y_{n})}{\partial x}}$ and $\bar{\frac{{\partial Z}_{i}\left( x_{n},y_{n} \right)}{\partial y}}$ are the averaged $x$ and $y$ derivatives of the $i^{th}$ Zernike polynomial. Collecting the local wavefront slopes from all $n$ droplets into a single vector $\vec{D}$ yields

$$\begin{aligned} \vec{D}=\nabla\vec{Z}\vec{a},\#\left( 16 \right) \end{aligned}$$

with

$\begin{aligned} \vec{D}=\left[ \begin{matrix} \Delta x_{1}/f_{drop} \\ \Delta x_{2}/f_{drop} \\ \begin{matrix} \vdots\\ \begin{matrix} \Delta x_{n}/f_{drop} \\ \begin{matrix} \Delta y_{1}/f_{drop} \\ \Delta y_{2}/f_{drop} \\ \begin{matrix} \vdots\\ \Delta y_{n}/f_{drop} \end{matrix} \end{matrix} \end{matrix} \end{matrix} \end{matrix} \right], \vec{a}=\left[ \begin{matrix} a_{1} \\ a_{2} \\ \begin{matrix} \vdots\\ a_{n} \end{matrix} \end{matrix} \right]\#\left( 17 \right) \end{aligned}$

and

$$\begin{aligned} \nabla Z=\left[ \begin{matrix} \frac{\bar{{\partial Z}_{1}\left( x_{1},y_{1} \right)}}{\partial x} & \frac{\bar{{\partial Z}_{2}\left( x_{1},y_{1} \right)}}{\partial x} & \begin{matrix} \cdots& \frac{\bar{{\partial Z}_{n}\left( x_{1},y_{1} \right)}}{\partial x} \end{matrix} \\ \frac{\bar{{\partial Z}_{1}\left( x_{2},y_{2} \right)}}{\partial x} & \frac{\bar{{\partial Z}_{2}\left( x_{2},y_{2} \right)}}{\partial x} & \begin{matrix} \cdots& \frac{\bar{{\partial Z}_{n}\left( x_{2},y_{2} \right)}}{\partial x} \end{matrix} \\ \begin{matrix} \vdots\\ \frac{\bar{{\partial Z}_{1}\left( x_{n},y_{n} \right)}}{\partial x} \\ \begin{matrix} \frac{\bar{{\partial Z}_{1}\left( x_{1},y_{1} \right)}}{\partial y} \\ \frac{\bar{{\partial Z}_{1}\left( x_{2},y_{2} \right)}}{\partial y} \\ \begin{matrix} \vdots\\ \frac{\bar{{\partial Z}_{1}\left( x_{n},y_{n} \right)}}{\partial y} \end{matrix} \end{matrix} \end{matrix} & \begin{matrix} \vdots\\ \frac{\bar{{\partial Z}_{2}\left( x_{n},y_{n} \right)}}{\partial x} \\ \begin{matrix} \frac{\bar{{\partial Z}_{2}\left( x_{1},y_{1} \right)}}{\partial y} \\ \frac{\bar{{\partial Z}_{2}\left( x_{2},y_{2} \right)}}{\partial y} \\ \begin{matrix} \vdots\\ \frac{\bar{{\partial Z}_{2}\left( x_{n},y_{n} \right)}}{\partial y} \end{matrix} \end{matrix} \end{matrix} & \begin{matrix} \begin{matrix} \begin{matrix} \begin{matrix} \begin{matrix} \ddots& & \end{matrix} & \vdots& \end{matrix} & \end{matrix} & \end{matrix} \\ \begin{matrix} \cdots& \frac{\bar{{\partial Z}_{n}\left( x_{n},y_{n} \right)}}{\partial x} \end{matrix} \\ \begin{matrix} \begin{matrix} \cdots& \frac{\bar{{\partial Z}_{n}\left( x_{1},y_{1} \right)}}{\partial y} \end{matrix} \\ \begin{matrix} \cdots& \frac{\bar{{\partial Z}_{n}\left( x_{2},y_{2} \right)}}{\partial y} \end{matrix} \\ \begin{matrix} \begin{matrix} \begin{matrix} \begin{matrix} \begin{matrix} \ddots& & \end{matrix} & \vdots& \end{matrix} & \end{matrix} & \end{matrix} \\ \begin{matrix} \cdots& \frac{\bar{{\partial Z}_{n}\left( x_{n},y_{n} \right)}}{\partial y} \end{matrix} \end{matrix} \end{matrix} \end{matrix} \end{matrix} \right]\#\left( 18 \right) \end{aligned}$$

Finally, the Zernike coefficients are obtained via the pseudoinverse of $\nabla Z$:

$$\begin{aligned} \vec{a}=\left( \nabla Z \right)^{\dagger}\vec{D},\#\left( 19 \right) \end{aligned}$$

This complete process yields the Zernike coefficients that describe the wavefront aberrations, thereby allowing a comprehensive reconstruction of the phase profile across the LC droplet array.

**Device-level mitigation of polarization-dependent geometric phase**

In addition to the dynamic (optical-path) phase responsible for microlens-based wavefront sensing, a polarization-dependent geometric phase may arise from the spatial variation of optical anisotropy within each droplet. To mitigate its influence at the hardware level, a polarization-resolved detection scheme may potentially be implemented in future work. For example, the transmitted optical field may be separated into two orthogonal polarization channels (e.g., using a polarization beam splitter), and the phase in each channel reconstructed independently. Since the dynamic optical-path contribution is polarization-independent, while the geometric phase is polarization-dependent, appropriate combination of the two channels may, in principle, reduce or isolate the geometric contribution.

We note that this approach represents one possible strategy, and its effectiveness would depend on the detailed polarization response of the droplet array. Further investigation is required to validate whether such channel combination can fully suppress the geometric phase under general conditions.

**Supplementary Note 3:** **Design considerations for droplet diameter and spacing**

**Droplet diameter**

The droplet diameter was selected by balancing polarization measuring robustness and spatial phase sampling density. Reliable Stokes reconstruction requires sufficient retardance diversity to provide suitable contrast for accurate Stokes reconstruction. Under 633 nm illumination, droplets with diameters below approximately 330 µm exhibited reduced modulation depth and degraded conditioning of the instrument matrix (see **Supplementary Fig. 1**), leading to unstable Stokes reconstruction. Therefore, 330 µm represents the minimum passive diameter ensuring robust full-Stokes retrieval in the present configuration.

From the wavefront-sensing perspective, however, smaller droplets are intrinsically advantageous. Reducing the droplet diameter increases the spatial sampling density across the effective pupil and correspondingly increase the maximum retrievable Zernike order in Shack-Hartmann reconstruction. In our configuration (effective pupil ≈ 2 mm), decreasing the droplet size would proportionally increase the number of sampling elements across the aperture and enable retrieval of higher-order aberrations.

The selected diameter therefore reflects a practical compromise between polarization-conditioning requirements and phase-sampling resolution. We note that this choice is specific to the present proof-of-concept system and does not represent a fundamental limitation. With further optimization of material birefringence, droplet geometry, or even active tuning strategies, smaller droplets could be employed while maintaining sufficient retardance, thereby improving spatial sampling density in future implementations.

**Inter-droplet spacing**

From a sampling perspective, reducing the edge-to-edge gap increases the fill factor of the array and maximizes the effective measurement aperture. In practice, however, excessively small separation increases the probability of droplet coalescence during fabrication and handling, arising from mechanical vibration, capillary interaction, and minor wetting variations on the alignment layer^[1]^. Such merging events distort the intended spherical-cap geometry and degrade both polarization modulation uniformity and microlens focal quality.

Based on repeated printing trials under identical process conditions, we determined that a spacing of approximately 10 µm represents the minimum reliable separation that preserves structural integrity while maintaining near-maximal packing density. This spacing is primarily limited by fabrication stability rather than optical design considerations. Future improvements in printing control, substrate engineering, or encapsulation strategies may enable further reduction of inter-droplet spacing, leading to higher packing density and enhanced sensing performance.

Accordingly, the final design adopts a droplet diameter of 330 µm and an edge-to-edge gap of 10 µm, representing a practical balance between polarization robustness, phase sampling density, and fabrication stability. These parameters therefore represent an initial design point, and further optimization is expected in future developments.

**Supplementary Note 4: Experimental set up**

To further demonstrate that the LC droplet array can operate on different wavelengths at the same time, we implemented a dual-wavelength experimental configuration in which each beam carries an independently defined polarization and phase state. It is important to note that compact optical sensing platforms already exist for individual modalities, including division-of-focal-plane polarimeters^[2]^, Shack–Hartmann wavefront sensors^[3]^, metalens-based Hartmann arrays^[4]^, integrated photonic polarimeters^[5]^, and MEMS-based adaptive optics devices^[6]^. However, many of these platforms are primarily designed for one function only and/or they require the use of complex structures and wavelength-specific designs to achieve multifunctionality.

By contrast, in the present LC droplet array, polarization modulation and microlens-based phase sampling are combined within the same droplet element, enabling simultaneous full-vectorial field sensing within a simple and low-cost platform. This co-localized mechanism naturally supports operation across multiple wavelengths without requiring structural redesign. Importantly, because the modulation relies on bulk birefringence rather than wavelength-specific resonances, both polarization and phase sensing remain stable and well-conditioned across different wavelengths, rather than being confined to a narrow design band.

The experimental setup utilized is shown in **Supplementary Fig. 3**, a 633 nm helium-neon laser (HNLS008L-EC, Thorlabs) is used as one light source, emitting a vertically polarized beam. Directly after the laser, a half-wave plate (HWP) (WPHSM05-633, 633 nm, Thorlabs) rotates the linear polarization to $0^{\circ}$, ensuring that the beam aligns properly with the LC inside the SLM (Hamamatsu LCOS-SLM X15213-01) afterwards. This alignment is crucial for achieving high modulation efficiency, as the SLM’s phase control depends on the polarization being parallel to the LC director’s fast axis. Once the polarization is set, the beam is expanded by a 4F system composed of a $10\times$ objective (RMS10X, 0.25 NA, Thorlabs) and a lens of 100mm focal length (AC254-100-A-ML, *f* = 100 mm, ARC: 400-700 nm, Thorlabs). This 4F arrangement enlarges the beam’s cross-section, ensuring uniform illumination across the SLM’s active area. The beam then reflects off two mirrors (BB03-E02, 400-750 nm, Thorlabs) to maintain a small incident angle on the SLM (less than $10^{\circ}$), with approximately $5^{\circ}$ of deflection. Keeping the incident angle minimal is important for uniform phase modulation and minimal distortion.

The SLM is programmed to introduce known phase aberrations, often represented by Zernike modes, each with an adjustable amplitude. By controlling these modes, the system can verify its phase measurement accuracy by applying specific wavefront distortions. After the SLM imposes the desired phase pattern, a second 4F system (AC254-100-A-ML, *f* = 100 mm, ARC: 400-700 nm, Thorlabs) relays the modulated wavefront to the next stage of the setup. The 4F configuration precisely maps the pupil plane from the SLM onto the droplet array, ensuring that the phase aberrations remain accurately preserved for analysis. The next critical component in the setup is the PSG, which is used during calibration. The PSG consists of a polarizer (LPVISC050, 510-800 nm, Thorlabs) and a QWP (WPQSM05-633, 633 nm, Thorlabs) that together create a range of controlled SoPs needed to calibrate the system matrix $A$. Once calibration is complete, the PSG is replaced by a four-division waveplate (FWP) (HWP: WP280, 400-700 nm, Edmund Optics; QWP: WP140HE, 400-700 nm, Edmund Optics) that modulates the beam’s polarization across its cross-section, thereby generating spatially varying polarization patterns to test the system’s polarization sensing ability.

A 532 nm green laser (532 nm DPSS Pointing Laser, 10 mW, Edmund) is introduced alongside the existing 633 nm red source (HNLS008L-EC, Thorlabs). For the green-light path, phase modulation is introduced using a wedge cell filled with nematic LC (E7, Synthon Chemicals Ltd.), which induces a tilt aberration due to its refractive index gradient. Since this modulation method differs from the red-light path, it provides a direct validation that the droplet array can simultaneously detect different phase distortions at different wavelengths. The green beam then passes through a separate FWP, encoding distinct SoPs across different regions.

Both beams, carrying independently modulated phase and polarization information, are combined at a beam splitter (BS043, 400-700 nm, Thorlabs) and directed onto the droplet array for simultaneous sensing. In the green and combined beam paths, pairs of mirrors were used in place of single reflections to keep the incidence angles shallow and avoid reflections exceeding $45^{\circ}$. To ensure that a clearly magnified image of the droplet array is obtained, a second 4F system, comprising of a $10\times$ objective (RMS10X, 0.25 NA, Thorlabs) and a 100 mm focal length lens (AC254-100-A-ML, *f* = 100 mm, ARC: 400-700 nm, Thorlabs), is used after the LC droplet array. This 4F system not only enlarges the image but also matches the droplet’s natural numerical aperture of approximately 0.3-this value follows directly from the droplet’s 330 µm diameter and its $\sim$0.55 mm focal length ($NA\simeq D/\left( 2f \right)\simeq0.33 mm/(2 \times0.55 mm)\approx0.30$ when $D\ll2f$).

Subsequently, a beam splitter (BS043, 400-700 nm, Thorlabs) divides the beam into two paths. In the first path, the beam passes through a polarizer set at $0^{\circ}$ and is detected by an RGB camera (Basler Ace acA4024-29uc, Edmund Optics), which naturally separates the red and green channels, enabling independent detection of their respective polarization information in a single measurement. The intensity information captured in each droplet image is used to reconstruct the incident beam’s Stokes vector via the calibrated system matrix $A$. In the second path, the beam is relayed through another 4F system and directed to a separate RGB camera (Basler Ace acA4024-29uc, Edmund) that records the focal spots produced by each LC droplet. By analysing the displacements of these focal spots between an unaberrated wavefront and an aberrated wavefront, local wavefront slopes are determined, enabling precise phase reconstruction based on Zernike coefficients.

The multi-wavelength capability of the present platform does not arise from wavelength-selective resonances, but from the fact that the LC droplets remain optically active for different wavelengths through the same birefringent modulation mechanism. The LC droplets function as bulk birefringent elements whose retardance scales smoothly with wavelength according to $\Delta\varphi\propto\Delta n\cdot t/\lambda$. Because the modulation arises from continuous anisotropic refractive index contrast rather than spectrally discrete resonances, each wavelength component is modulated by the same droplet through the same physical mechanism.

**Prospective extension toward wavelength-sensitive operation**

In the present work, dual-wavelength operation was demonstrated as a proof-of-principle, confirming that different spectral components can be analyzed simultaneously within a single droplet array without structural redesign. For the nematic LC E7 droplets used here, the wavelength dependence of the birefringence within the visible range is moderate, resulting in only subtle variations in transmitted polarization patterns and POM textures between 532 nm and 633 nm illumination.

More pronounced spectral sensitivity could potentially arise in materials exhibiting stronger dispersion of the birefringence or enhanced electric field-tunable retardance. In such cases, wavelength-dependent variations in the spatial intensity or polarization distribution may become more evident, suggesting the possibility that spectral information could be indirectly encoded in the transmitted optical field.

At the present stage, this concept remains exploratory, and its practical implementation would depend on the extent to which wavelength-dependent features can be robustly distinguished under realistic conditions. Nevertheless, this observation indicates a possible route toward extending the droplet platform beyond polarization and phase sensing, potentially enabling additional functionality within a unified architecture. A systematic investigation of this direction is the subject of ongoing work and will be reported in future studies.


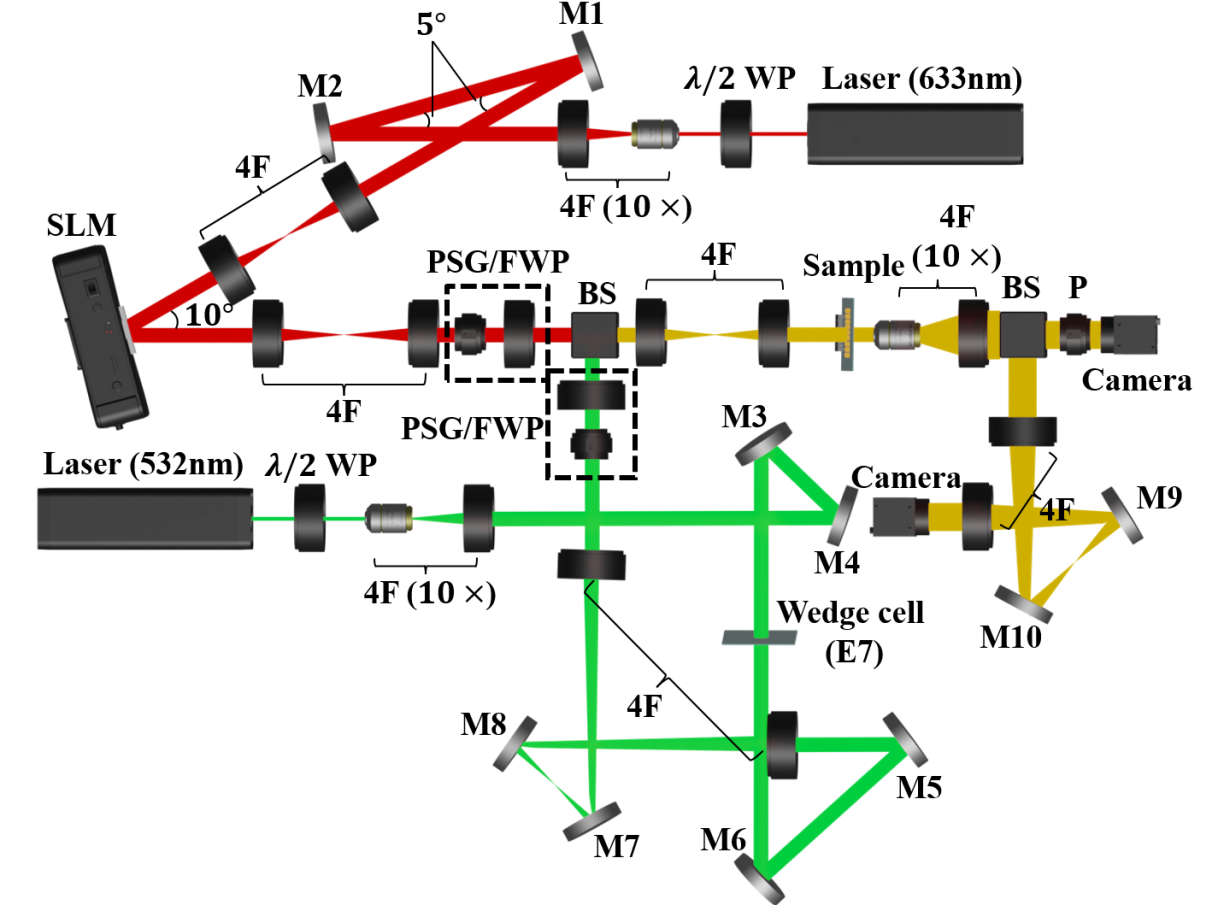


**Supplementary Figure 3 Schematic diagram of experimental configuration for the multi-wavelength vectorial field sensing system.** A 633 nm laser beam (red) is first polarization‑aligned by a HWP and then phase‑modulated by an SLM via a $10^{\circ}$ incidence. Mirrors M1 and M2 relay the modulated beam through a 4F system before it enters the PSG/FWP, while the 532 nm green beam is modulated by a wedge cell filled with nematic LC (E7) and another FWP. The beams are combined and incident on a LC droplet array, after which a non‑polarizing beam splitter divides the transmitted light into two arms. Two RGB cameras capture the output to independently extract phase and polarization information for both wavelengths. In the polarization arm, the droplet‑array intensity is imaged through a fixed polarizer onto an RGB camera for Stokes vector reconstruction. In the phase arm, a second 4F relay the array’s focal‑spot pattern onto another RGB camera for Shack-Hartmann-based wavefront analysis. To maintain reflection angles below $45^{\circ}$, the green beam path and the combined beam path employ pairs of mirrors (M3–M10, respectively) instead of single reflections.

**Supplementary Note 5: SLM calibration**

Accurate phase reporting in the main experiments requires a direct, quantitative link between the dimensionless “amplitude” value entered in the SLM control software and the physical RMS OPD that the device introduces to the wavefront. Without calibration, every Zernike coefficient quoted in the experiment would float on an arbitrary scale and could not be reproduced. We therefore performed an external calibration that compares the SLM output with an independently traceable reference: a commercial SHWS. The experimental layout follows a conventional configuration for phase calibration (**Supplementary Figure 4**). After reflecting from the SLM, the collimated beam enters the SHWS, where a commercial lenslet array (Edmund Optics 15-812) with 300 µm pitch and 18 mm focal length samples the wavefront and forms a grid of focal spots. To accurately relay this focal plane onto the camera, a 4F imaging system is placed directly after the lenslet array.


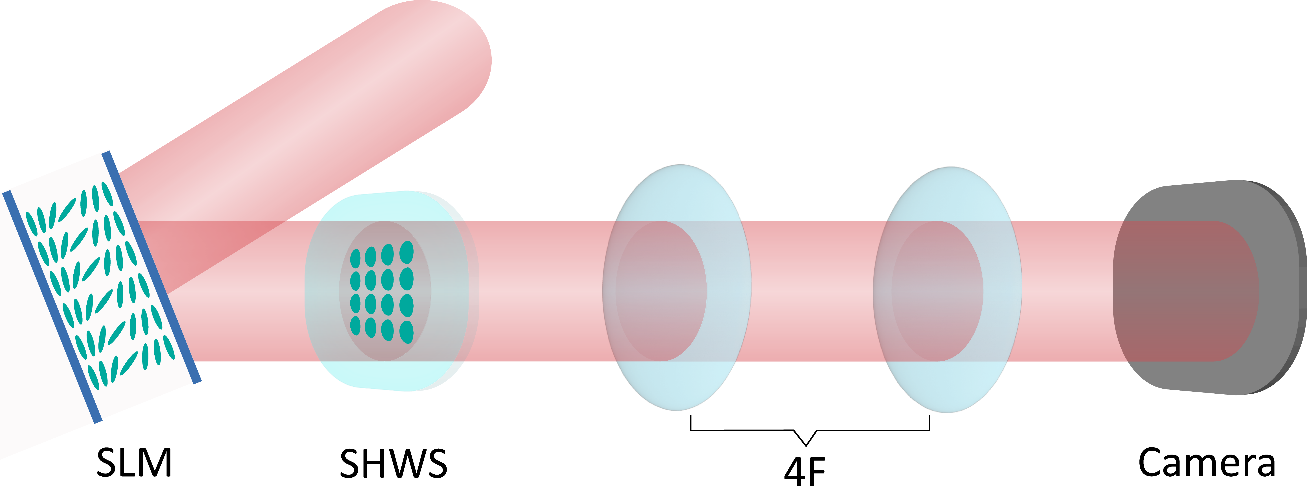


**Supplementary Figure 4 Schematic of the experimental arrangement for calibrating the SLM using a SHWS.** A collimated beam is phase-modulated by the SLM and then directed into the SHWS, where a commercial lenslet array (300 µm pitch, 18 mm focal length) samples the wavefront and forms a grid of focal spots. A 4F imaging system relays the focal plane onto a CCD camera for accurate measurement of spot displacements and wavefront reconstruction.

Calibration begins by applying a uniform phase (zero modulation) to the SLM, allowing the SHWS to record the baseline wavefront of the system. This reference map captures any intrinsic aberrations or alignment offsets, which are subsequently subtracted from all measured phase profiles to ensure accurate reconstruction. Next, for each Zernike mode of interest, a sequence of five amplitude values-2, 5, 10, 15, and 20-is applied. At each setting, the SLM generates a corresponding phase profile, and the RMS OPD is extracted from custom wavefront reconstruction code for each amplitude value. Each set of five measurements is fitted using linear least squares regression, yielding a mode-specific calibration coefficient $k_{n}$ that converts software amplitude into RMS OPD. For example, horizontal and vertical tilt exhibit slopes of approximately $0.0165$μm/unit, while defocus and astigmatism yield slightly different slope values, reflecting the distinct spatial energy distributions of each Zernike mode (**Supplementary Figure 5**). These calibration coefficients are stored in the SLM control software to ensure that each commanded phase aberration corresponds to a physically defined and reproducible RMS OPD value. The calibrated SLM output is then used as the reference for the droplet-array-based wavefront measurement, enabling direct comparison between the generated and reconstructed phase profiles.


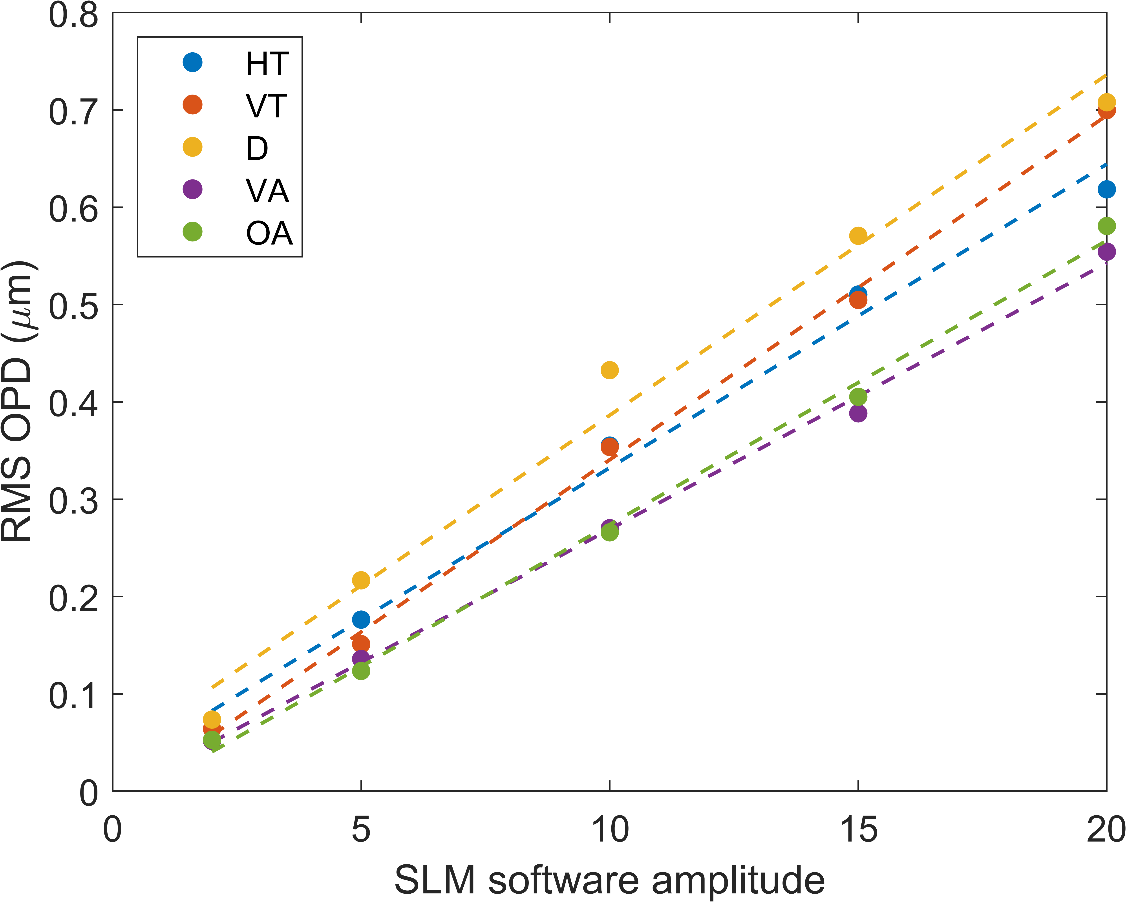


**Supplementary Figure 5 SLM calibration curves for five Zernike modes using a commercial SHWS.** Each curve shows the measured RMS OPD as a function of the SLM control software amplitude for a specific Zernike mode: horizontal tilt (HT), vertical tilt (VT), defocus (D), vertical astigmatism (VA), and oblique astigmatism (OA). Data points represent wavefront measurements from a SHWS with a commercial lenslet array, and dashed lines indicate results following linear least-squares regression. The extracted slopes provide mode-specific calibration coefficients that convert SLM software input values into physically meaningful RMS OPD in microns.

**Supplementary Note 6: Phase response to scaled Zernike aberrations**

In the experiment, the SLM generated a circular aberration pattern with a diameter of 10 mm, while the diameter of the effective pupil at the LC droplet array was only 2mm, as determined by the aperture of the LC droplet array. To ensure that the intended phase aberration amplitudes were accurately applied within this smaller pupil, a scaling procedure is implemented based on the radial dependence of Zernike modes. Because Zernike polynomials are orthogonal over a unit circular aperture and their amplitude scales with the radial location within this unit aperture, the effective amplitude within a reduced pupil size must be compensated accordingly. Specifically, if the full SLM aperture is defined as having a normalized radius of 1, then the LC droplet array occupies only 0.2 of this radius (i.e., $\frac{2 mm}{10 mm}=0.2$). Therefore, for Zernike modes of different radial orders, a scaling factor proportional to the inverse of this radius raised to the power of the mode’s radial order is applied. For instance, first-order modes, such as tilt, scale linearly with radius and were therefore multiplied by $\frac{1}{0.2}=5$, second-order modes, such as defocus or astigmatism, scale quadratically and were multiplied by $\frac{1}{\left( 0.2 \right)^{2}}=25$.


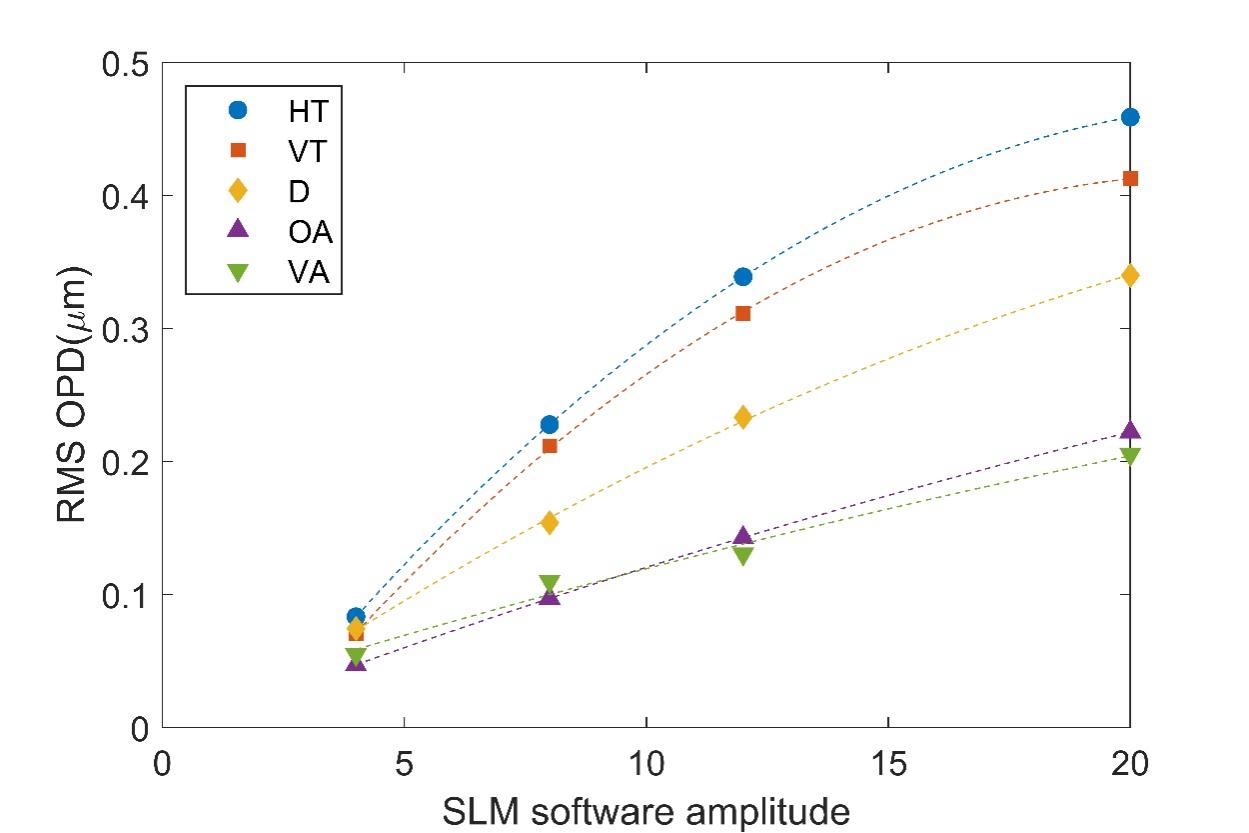


**Supplementary Figure 6 Measured RMS OPD response of the LC droplet array phase sensor to different Zernike modes applied by the SLM.** The *x*-axis represents the effective SLM amplitude after applying radius-dependent scaling, ensuring accurate generation of Zernike amplitudes within the 2 mm pupil covering the droplet array. The y-axis shows the reconstructed RMS OPD measured by the system. Results are shown for horizontal tilt (HT), vertical tilt (VT), defocus (D), oblique astigmatism (OA), and vertical astigmatism (VA). Dashed lines represent quadratic fits to the data.

To incorporate this scaling into actual measurements, we combined the radius-dependent compensation factor with the mode-specific calibration coefficients obtained from the SLM calibration procedure. This ensured that for each Zernike mode, the applied software amplitude on the SLM could be accurately converted into a physically meaningful RMS OPD within the reduced 2 mm pupil defined by the droplet array. Using this approach, we programmed the SLM to apply a series of Zernike aberrations at various amplitudes, covering both first-order (tilt) and second-order (defocus and astigmatism) modes. The phase response was then measured using the LC droplet array sensor, and the reconstructed RMS OPD values were plotted as a function of the effective SLM amplitude after radius scaling, as shown in **Supplementary Figure 6**.

The results demonstrate that the measured phase increases with the input amplitude for all modes tested. While the overall trends agree with theoretical expectations, a mild nonlinearity is observed, particularly at higher amplitudes. This deviation is likely caused by the SLM approaching its modulation limit, where the phase response no longer scales linearly with the software input due to the saturation of the LC’s phase modulation range. Nonetheless, the measured trends across different Zernike modes confirm that our scaling model well predicts the phase amplitudes within the system’s operating range.

**Supplementary Note 7: Director-field simulation and optical reconstruction**

To establish a direct structure–property relationship, we performed numerical simulations of the equilibrium director configuration based on the full Frank–Oseen elastic continuum theory without adopting the one-constant approximation. The total elastic free energy density is given by:

$$\begin{aligned} F_{elastic}=\frac{1}{2}K_{11}\left( \nabla\cdot\vec{n} \right)^{2}+\frac{1}{2}K_{22}\left( \vec{n}\cdot\left( \nabla\times\vec{n} \right) \right)^{2}+\frac{1}{2}K_{33}\left( \vec{n}\times\left( \nabla\times\vec{n} \right) \right)^{2} \#\left( 20 \right) \end{aligned}$$

where $K_{11}$​, $K_{22}$, and $K_{33}$​ denote the splay, twist, and bend elastic constants, respectively. Homeotropic anchoring boundary conditions were imposed at both the substrate and LC–air interfaces. The equilibrium director field was obtained by minimizing the total free energy, yielding the radially symmetric configuration shown in **Supplementary Fig. 7**.


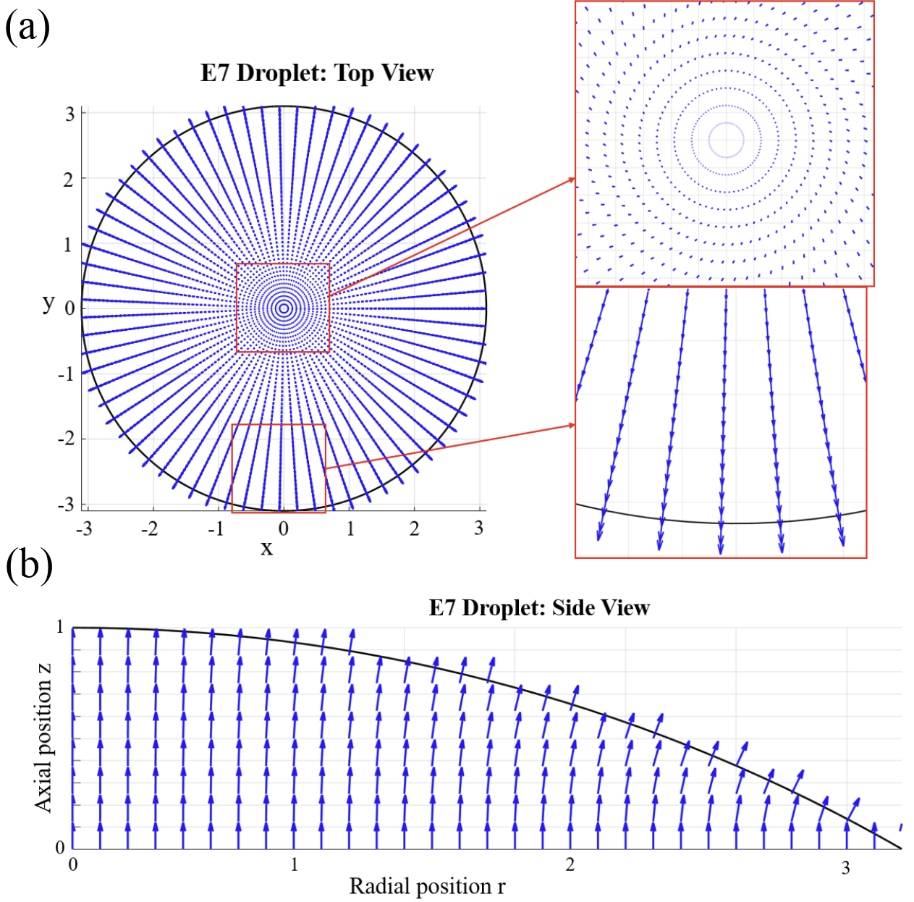


**Supplementary Figure 7: Simulated director field of a printed nematic LC (E7) droplet on a homeotropic alignment layer.** (a) Top view: director at the centre is vertically aligned, transitioning smoothly to a radial orientation near the surface of the droplet due to the perpendicular anchoring at the LC-air interface. Insets show detailed transitions at the centre and edge. (b) Side view: the director tilts progressively from a vertical orientation at the centre to being more radial near the droplet surface (LC-air interface).

From the simulated director field $n(r,\phi,z)$, the optical response was reconstructed by extracting the in-plane projection of the director to determine the local fast-axis orientation, and by integrating the effective birefringence along the optical axis to obtain the spatial retardance distribution.

The reconstructed retardance profile exhibits a radial increase from the droplet centre, reaching a maximum at intermediate radius and decreasing toward the edge, consistent with the experimentally retrieved Mueller-matrix retardance maps (**Supplementary Fig. 1**). Similarly, the simulated fast-axis orientation displays azimuthal symmetry in agreement with polarization-resolved measurements.

This consistency confirms that the observed optical modulation directly originates from elastic-energy-minimized director configurations under homeotropic anchoring, thereby establishing a clear structure–property link between droplet morphology and polarization response.

Reference

1. Kamal, W., et al., *Electrically Tunable Printed Bifocal Liquid Crystal Microlens Arrays.* Advanced Materials Interfaces, 2020. **7**(16): p. 2000578.

2. Tu, X., et al., *Division of focal plane red–green–blue full-Stokes imaging polarimeter.* Applied Optics, 2020. **59**(22): p. G33–G40.

3. Platt, B.C. and R. Shack, *History and principles of Shack-Hartmann wavefront sensing*. 2001, SLACK Incorporated Thorofare, NJ. p. S573–S577.

4. Yang, Z., et al., *Generalized Hartmann-Shack array of dielectric metalens sub-arrays for polarimetric beam profiling.* Nature Communications, 2018. **9**(1): p. 4607.

5. Dong, J. and H. Zhou, *Polarimeters from bulky optics to integrated optics: A review.* Optics Communications, 2020. **465**: p. 125598.

6. Zhang, Y., S. Poonja, and A. Roorda, *MEMS-based adaptive optics scanning laser ophthalmoscopy.* Optics Letters, 2006. **31**(9): p. 1268–1270.
